# The Tulumbe! Partnership: a case study in developing a community-led research agenda to address HIV among African immigrants in the United States

**DOI:** 10.3389/fpubh.2024.1406397

**Published:** 2024-09-20

**Authors:** Chioma Nnaji, Lorraine Anyango, Carol Bova, Frederick Kiggundu, Mbita Mbao, Dara Oloyede, Ayomide Omotola

**Affiliations:** ^1^Africans For Improved Access Program, Multicultural AIDS Coalition, Boston, MA, United States; ^2^UMass Chan Medical School, Tan Chingfen Graduate School of Nursing, Worcester, MA, United States; ^3^Tulumbe! Project, Multicultural AIDS Coalition, Boston, MA, United States

**Keywords:** community-engaged research, HIV/AIDS, patient-centered, African immigrants, community engagement, refugees, migrant health

## Abstract

Grassroots, community organizations are trusted resources within communities, which puts them in an ideal position to effectively engage individuals impacted by health inequities in defining meaningful research priorities. A community-centered approach to HIV research is critical for African immigrants living in the United States, who experience stigma and other socio-structural barriers to HIV prevention, care, and research engagement. Supporting community organizations with financial resources and capacity building activities to lead the development of research agendas ensures better alignment with community interests and fosters sustainability. We developed a community-initiated and -led research engagement project—Tulumbe!, which prioritized community leadership in all project activities. Community forums, health care provider and community questionnaires, interviews, and report-back sessions were held to examine the research interests and health concerns voiced by African immigrants. The iterative, community-led engagement process of more than 200 African immigrants, health providers, and researchers resulted in a community-defined research agenda with six areas of focus: family communication; self-efficacy for African immigrant women; deconstructing masculinity for African immigrant men; sexual health education for African immigrant youth; HIV stigma; and health literacy. Time, resources, and flexibility are needed to develop a viable community-led research partnership. Investing in community leadership not only produced a patient-centered research agenda but also led to community ownership of the process and results; thus, all partners were committed to sustaining the work.

## 1 Introduction

Communities have been at the forefront of organizing and implementing strategies to respond to the HIV/AIDS crisis since it emerged in the 1980s (1). Efforts, often spearheaded by informal community networks and established community organizations, have historically emphasized advocacy and prioritized the needs of minoritized and racialized groups. Communities impacted by HIV—including people living with HIV, sex workers, people who inject drugs, transgender people, and gay men—were at the forefront of the battle, providing vital support, education, and advocating for HIV-friendly policies, as well as securing essential government funding. Initiatives such as the Minority AIDS Initiative were instrumental in funding the development of culturally and linguistically appropriate service models, thus significantly improving overall access to HIV prevention and care services (2).

An asserted shift toward more inclusive and community-centered research methodologies within the HIV response has been essential to advancements in the field of HIV research (3–5). Approaches such as Community-Based Participatory Research (CBPR) and Community-Engaged Research (CER) have aimed to bridge the gap between translating research findings to action for social change that improves community health and eliminates health inequities. CBPR/CER are ongoing, iterative processes that prioritize the active involvement of community members in every phase of the research process, ensuring that research is informed by community perspectives and aligns with community norms, values, and interests (6, 7). CBPR/CER relationships are built on trust, reciprocal learning, multi-directional capacity building, and the establishment of equitable power structures and processes (8). Most critical to a CBPR/CER process is the beginning, where communities define the research agenda to fully align with health priorities.

The importance of community engagement is further underscored by the ‘Ending the HIV Epidemic’ (EHE) strategy for the United States, which emphasizes the importance of community-driven solutions to leverage scientific advances in HIV prevention, diagnosis, treatment, and outbreak response. A key aspect of this strategy is mandating each jurisdiction under EHE to allocate a minimum of 25% of their EHE funds to programs and initiatives that promote community engagement. These initiatives are to be led by community-based organizations, reinforcing the central role of communities in driving the HIV response (1, 9).

There are various participatory methods for CBPR/CER (10). Across HIV research, ranging from epidemiological studies to clinical trials, community advisory boards (CABs) have emerged as effective tools for meaningfully engaging the community (4, 11). These boards consist of community members and representatives from diverse organizations connected to the issue. They serve as a valuable mechanism for seeking community guidance throughout the research process, from setting research priorities to the development of research questions to the dissemination of findings.

However, while community engagement practices like advisory boards have shown significant improvements in transparency and inclusion, there are still existing barriers and limitations that not only hinder effectiveness (12) but also reinforce injustices. There is a potential mismatch between the research agenda set by community members and researchers, stemming from differences in expertise, priorities, and perspectives. Moreover, unequal decision-making authority between researchers and community members may hinder genuine collaboration. Challenges in resource allocation, time constraints, and logistical issues can impede effective engagement processes. Research shows that community-based organizations often lack the necessary research literacy to engage as equal research collaborators, yet they demonstrate a strong willingness to enhance their capacity and actively participate as full partners in research endeavors (13). Existing barriers and limitations perpetuate epistemic injustices, wherein the knowledge, perspectives, and expertise of minoritized and racialized groups may be undermined, disregarded, not given equal weight, or seen as less credible compared to those of academic research partners or other socially privileged members within the CBPR/CER partnership (14). Ongoing epistemic injustices hinder community involvement in deciding research goals, perpetuating mistrust rooted in historical and current breaches of autonomy and medical abuses.

One CBPR/CER strategy that warrants attention is directly funding community-based organizations (CBOs) to lead community engagement efforts throughout the research process, starting from the generation of ideas for research projects or studies. CBOs’ familiarity with and proximity to communities position them to not only engage individuals impacted by HIV inequities in shaping meaningful research but also to enhance research literacy and foster community ownership of the work. However, despite their critical role, CBOs face challenges in securing funding and establishing the infrastructure required by funders (1). They must unfairly compete with larger, well-established research or academic institutions. The complex and labor-demanding grant application procedures necessitate substantial documentation based on Western models of knowledge creation. Compliance with the criteria to be lead personnel is often biased toward traditional academic qualifications. This becomes increasingly unjust given the limited availability of funding for community-engaged research efforts. Funding agencies may not consistently prioritize community interests or recognize the transformative potential of involving CBOs in decision-making processes related to research agendas. These barriers underscore the pressing need for a decolonized research enterprise, which recognizes and values multiple forms of knowledge and actively seeks to dismantle traditional hierarchies in knowledge production. This shift promotes equitable opportunities for community-led initiatives and offers more accessible and flexible funding structures for diverse CBOs (15).

Our key argument emphasizes that CBOs are well-positioned and capable of leading efforts to define research agendas. The effective engagement strategies implemented by CBOs cater to the cultural and linguistic norms of communities. A community-led approach enhances the relevance and impact of research by aligning with the lived experiences and needs of communities. This article documents our process and outcomes, making a significant contribution to the literature on effective community engagement strategies for racialized and minoritized populations. Additionally, it highlights the need for a pivotal shift in the research enterprise.

## 2 Context

Shifting to a more equitable approach is especially critical in the context of HIV research among marginalized populations. African immigrants are one of the fastest-growing immigrant communities in the United States and accounted for the fastest growth in the US Black immigrant population (16). Between 2010 and 2018, the African immigrant population increased by 52%, while the overall immigrant population only grew by 12% during that time (17). Although more research is needed, studies have begun to highlight the unique HIV needs of the population. African immigrants account for a disproportionate number of individuals living with HIV (18). They are less likely to test for HIV and more prone to delayed engagement in care compared to other Black communities and US-born people (19). This is due to intersecting structural barriers, such as stigma and unique challenges rooted in anti-Black, anti-immigrant discrimination (20, 21). These barriers create fear and mistrust limiting engagement in HIV prevention and care, as well as research.

One of the strengths of the African immigrant community in the United States is the trusted sources that have the cultural knowledge, skills, and expertise to break down barriers. African immigrants tend to form close-knit, ethnic enclaves consisting of faith and civic organizations founded by earlier immigrants. Many of these organizations serve as a place to maintain cultural ties, as well as provide support and other services. These truly grassroots entities are often the only agents that African immigrants trust and utilize regularly, putting them in a unique position to lead research engagement.

The term ‘stakeholders’ carries a historical and harmful representation of colonization and the stealing of land endured by Indigenous peoples. Hence, we use different terms to describe organizations or individuals with an interest or concern in addressing HIV among African immigrants. Terms such as ‘interested parties,’ ‘partners,’ or ‘collaborators’ depict the nature of our engagements without causing discomfort or perpetuating historical injustices. ‘Stakeholder’ is only used when presented as part of a title for a document or formal statement.

## 3 Key programmatic elements

### 3.1 Project background

The Africans for Improved Access (AFIA) program at the Multicultural AIDS Coalition (MAC) has a strong history of successfully providing HIV/STD outreach, education, testing, and navigation services to African immigrants in Massachusetts (22, 23). AFIA’s engagement strategies aim to support long-standing community relationships, destigmatize HIV and related topics, build trust, and dispel myths that inhibit the community from seeking services (24). Research done “by and with” the community is seen by MAC and its programs as critical to mobilizing individuals most impacted by HIV/AIDS in defining shared priorities for improving service delivery and relevant public policy to effect change. Because of this, staff sought opportunities to engage in research projects that are directly led by AFIA, its partners, and leaders in the community.

The Patient-Centered Outcomes Research Institute (PCORI) funded AFIA through its Pipeline to Proposal (P2P) Awards to establish and build capacity for a patient-centered research partnership addressing HIV among African immigrants. PCORI was formed in 2010 to support patients and their caregivers in making informed decisions about clinical care. The institute funds multi-year patient-centered engagement and comparative effectiveness research (CER) projects. Patient-centered means prioritizing the involvement of patients in the design, implementation, analysis, and dissemination phases. Patients are defined as individuals, “… who are representative of the population of interest in a study, as well as their family members, caregivers, and the organizations that represent them (25).”

As part of its patient engagement portfolio, PCORI launched the Pipeline to Proposal (P2P) Awards, which aimed to engage individuals and groups not typically involved in clinical research. The program was a three-tier funding mechanism designed to build the capacity of patient-centered partnerships to develop comparative effectiveness research questions that could be translated into research proposals for PCORI funding or other health research funders. Tier I focused on building partnerships and capacity for patient-centered research, Tier II supported partnerships in developing research questions, and Tier III provided funding to develop a full research proposal ready for submission to PCORI or other funding sources. In addition to small funding, grantees were provided training and technical assistance through webinars, one-on-one consultations, and tools/templates (26). Because individuals or groups could receive the funding without a university, researcher, or incorporated entity, grassroots community-based organizations were able to directly apply and lead in the development of a partnership, as well as lead engagement to form community-defined research directions.

AFIA was funded for Tier I (2016) and Tier II (2017). In 2018, funding for Tier II P2P grantees to progress through the pipeline to Tier III was discontinued. Throughout the funding cycle, the Tulumbe! Partnership (hereinafter referred to as Tulumbe!) met biweekly to plan and implement project activities. “Tulumbe!” is a Luganda (language spoken in Uganda) word that means, “to engage.” Table 1 lists the components of Tier I and Tier II funded by PCORI P2P including, the goals, funding amounts, and how the funding was used.

**Table 1:** Tulumbe! process and outcomes.

Components	P2P Tier I	P2P Tier II
Goal	Build a partnership and increase knowledge to develop a patient-centered CER project.	Strengthen the partnership and further develop the infrastructure, with the goal of refining the list of CER ideas to a single research question.
Funding Amount	$15,000	$25,000
Project Period	9-month term	12-month term
Activities	Biweekly partnership meetings Member skills and assets matrix Two community forums Provider questionnaire Dissemination	Biweekly partnership meetings Member recruitment Community report back forum Engagement meetings CER 101 workshop Community questionnaire Dissemination
PCORI Tools/Templates	Recruitment strategies CER ideas table	Stakeholder engagement plan CER questions table Communications plan
Deliverables	Name of partnership Partnership structure Governance document Five HIV-related research topics	Communications plan Stakeholder engagement plan Six HIV-related research topics
Community Leadership / Engagement	Project Lead and Coordinator represent the patient population and work at a patient advocacy organization Core circle members representing diverse interested parties with a majority being patient partners (first- or second-generation African immigrants) Patient partners led agenda items during partner meetings Patient partners led and engaged in participatory data analysis sessions Patient partners co-facilitated community forums	Same activities as Tier I, 1–4 Patient partners co-facilitated community report-back forum Patient partners identified outreach locations and administered questionnaires Patient partners disseminated outcomes Patient partners attended relevant health and HIV events

CER, Comparative Effectiveness Research; PCORI, Patient-Centered Outcomes Research Institute; P2P, Pipeline to Proposal Awards.

The overall purposes of the Pipeline to Proposal award were to develop a multi-sector research partnership, build capacity for patient-centered outcomes research, and inform a subsequent research application. In addition, the P2P award specifically prohibited the use of funds for research purposes. The information presented in this article is a retrospective evaluation of the project.

### 3.2 Tier I overview

During Tier I, Tulumbe! developed the partnership structure and engagement strategies for African immigrants and key organizations. We held two community forums to hear from African community members, leaders, and civic and faith organizations about barriers and facilitators to utilizing HIV services. Forums were divided into two parts—video with a large group discussion and small discussion groups by gender. Following the introduction, we showed a 25-min, locally developed video, In Our House: An African Story, which depicts an African immigrant family’s journey in dealing with HIV and HIV-related stigma in the United States. Participants were encouraged to discuss the video and ask questions. After lunch, participants were divided into gender-specific small discussion groups. Each group had two facilitators who prompted a discussion on ‘what works’ and ‘what does not work’ in accessing and utilizing HIV primary and secondary services. The facilitators took notes during the discussions. Both community forums lasted 3 h and ended with thanking the community members for their time and celebrating with an African dance and drumming performance. Participants received a $50 gift card for attending the community forums and providing valuable input. Testing for HIV, gonorrhea, chlamydia, syphilis, and hepatitis C was provided on-site by the AFIA program.

During Tier I we also administered a 17-item questionnaire to healthcare providers on the critical issues and successes experienced when providing HIV screening, linkage to care, and treatment services to African immigrants.

### 3.3 Tier II overview

Engagement activities for Tier II started with convening a community report-back forum (approximately 4 h) where community members, providers, and researchers prioritized 5 health topics defined in Tier I and began developing the content for research questions. At the forum, we provided a brief history of Tulumbe! and reported back on Tier I accomplishments. We also shared the HIV epidemiology data on the epidemic among African immigrant communities. All this information provided context for attendees to fully engage with a prioritization exercise. We continued to gain feedback on the health topics and ideas for research questions by presenting at various provider meetings. In addition, we distributed a questionnaire to have African immigrant community members prioritize CER topic areas and questions. Community members who completed the questionnaire received a $10 gift card as a token of appreciation for their time and valuable input.

### 3.4 Data analysis

The theoretical perspective that guided the project, including the data analysis, was grounded in a participatory approach. Our approach was informed by CBPR principles, which emphasizes active collaboration between community members, researchers, and other interested parties. Academic partners acknowledge power differentials and work to reduce these differentials by building trust, mutual respect, and community leadership.

Qualitative data were documented by notes taken during discussions and staff written reports. Questionnaire and evaluation data were collected in hard copy, assigned a unique identification number, and analyzed using Statistical Analysis System version 9.3 (SAS Institute, Cary, NC). Descriptive statistics were computed for each variable. All data were reviewed with Tulumbe! partners during in-person biweekly meetings. Partners engaged in an iterative, participatory data analysis process to identify themes and make meaning of the data (further described in Results section).

## 4 Results

### 4.1 Partnership development

Tulumbe! consisted of African immigrants living with HIV and representatives from key organizations in Massachusetts’ HIV services landscape, including clinical researchers, state public health officials, clinical social workers, direct care providers, advocates, and African immigrant community and faith leaders. Majority of the leadership team members were first- or second-generation African immigrants, representing several African countries, including Nigeria, Kenya, Uganda, Sierra Leone, and Liberia. In addition, most leadership team members had been involved in HIV care for at least 5 years, with several having over 10 years of experience. Community members and direct care providers on the leadership team were actively involved in their local communities, holding positions in African civic organizations, and participating in events. These deep community connections and extensive knowledge about providing HIV services to African immigrants supported effective communication about the project and the development of clear engagement strategies.

Early during Tier I, partners named the partnership “Tulumbe!” to reflect our vision.

Partners developed a governance document and created a partnership structure that centered around a continuous engagement process for African immigrants, providers, researchers, and relevant organizations. The document created agreement around the partnership’s mission, values, guiding principles, structure, responsibilities, and decision-making process, as seen in Table 2.

**Table 2:** Tulumbe! governance document.

Section	Brief description
Partnership Name	“Tulumbe!” is a Luganda (language spoken in Uganda) word that means, “to engage.” The name helped to form an identity and it became a mantra within the community. Tulumbe! is a call upon all partners, diverse organizations, and the general community to come together and engage in coming up with solutions that will address ending the HIV epidemic among African immigrants in Massachusetts.
Mission Statement	Our mission is to co-create a robust, sustainable partnership that considers the unique contributions of patients, community members, stakeholders, and researchers to reduce the negative impact of HIV among African immigrants through knowledge acquisition, stigma reduction, cultural awareness as well as engagement in care and services.
Values	We value Respect, Culture, Transparency, Collaboration, Diversity, and Community.
Guiding Principles	Our guiding principles are: (a) Ensuring that the African Immigrant Community is an active participant in reducing HIV-related stigma, encouraging HIV prevention, and improving the health of the community; (b) Developing a robust research partnership that values and respects diverse expertise; and (c) Creating evidence-informed solutions to improve the health and welfare of African immigrants in Massachusetts.
Organizational Structure and Responsibilities	The partnership includes Project Leadership, the Core Circle, the Circle of Engagement, the Circle of Champions, and our engagement with patients and the community.
Meeting Structure	Meetings will be held at least once per month throughout the project and at least one meeting during the interim period between P2P funding cycles.
Partnership Decisions	Decisions will be made by consensus of a quorum of the Core Circle after the issue is opened for debate and discussion.
Review of Governance Document	This governance document will be reviewed three times per year (October, January, and April) during the P2P initiative (Tier 1, Tier 2, and Tier 3).
Conflict of Interest	Core Circle members have a duty to disclose to project leadership the existence of any financial interest or conflict of interest that may arise during the project. A conflict of interest may exist when a person derives personal benefit from actions or decisions made in their official capacity as a member of the project team.
Inactive Members (added in Tier II)	Core Circle members who miss more than 4 scheduled meetings (unexcused absences) within 3 months and have not taken an active role in contributing to the work of the partnership will be asked to forfeit their position. There is a possibility to re-engage with the Core Circle later. Members who wish to voluntarily leave the partnership are welcome to do so by sending an email to the Project Lead and Project Coordinator.

**Figure 1:**
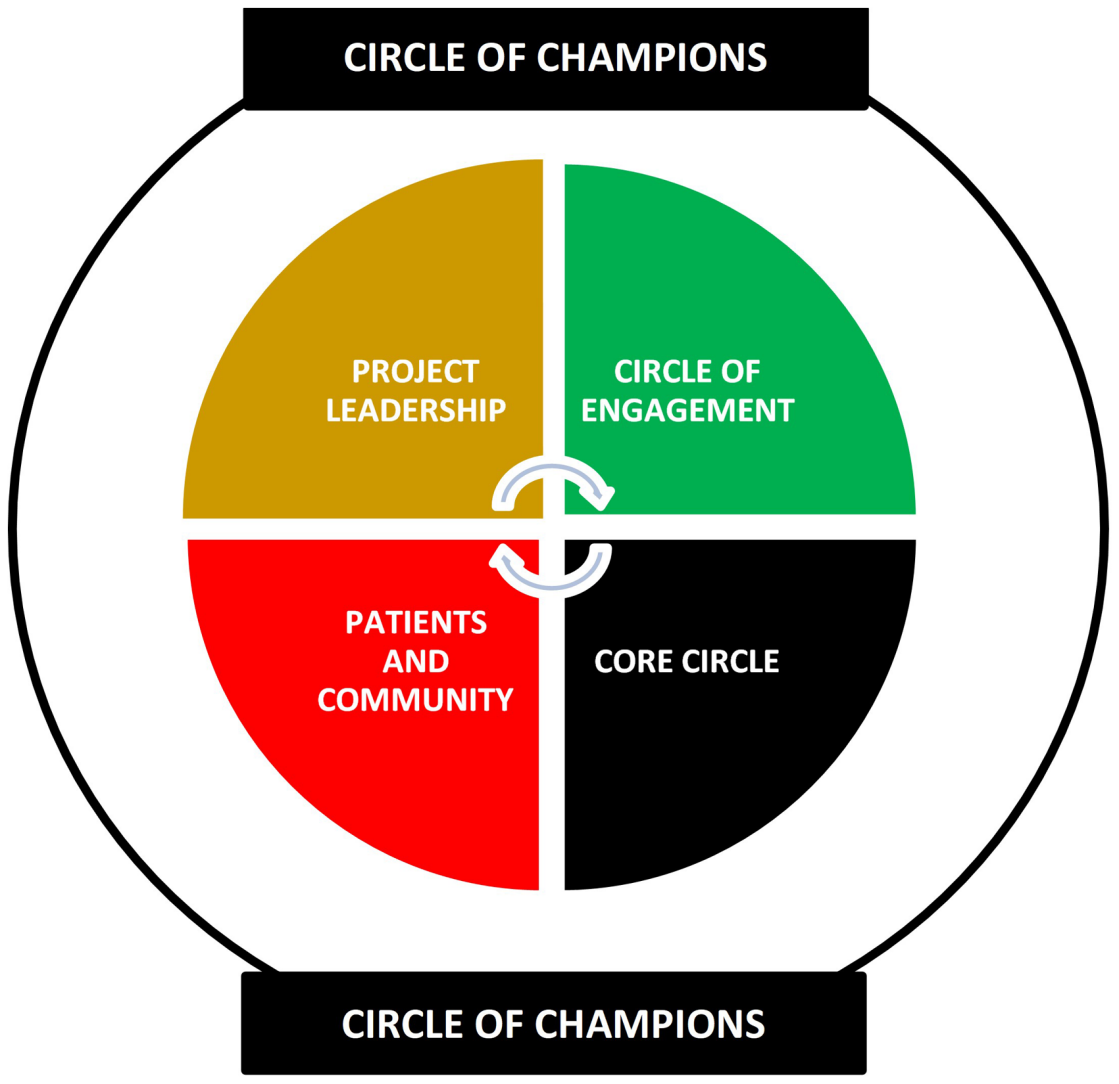
Tulumbe! partnership organizational structure.

As shown by Figure 1, Project Leadership, the Core Circle, the Circle of Engagement, patients, and the community had shared ownership of the project. Members of the Circle of Champions group were invested in the project and constantly served as influencers and advisors.

Most members of the Core Circle served voluntarily; however, African immigrant community members received a stipend for attending meetings which included meals. Childcare, parking, and travel were reimbursed separately.

### 4.2 Tier I results

Community Forums: Two community forums were held—one in Lowell, MA at a local African church and the other in Boston, MA at the AFIA office. African immigrants were recruited through flyers distributed via the social networks of leadership team members and through direct outreach to African-led organizations and community leaders by AFIA staff. Approximately 50 community members, including both men and women aged 18 years and older, were in attendance. Diverse African countries were represented including, Eritrea, Kenya, Liberia, Nigeria, Uganda, Zambia, and Zimbabwe.

For the small group discussions, participants were divided into gender-specific groups—men and women. This division fostered a comfortable and open environment, enabling participants to freely discuss sensitive topics, including HIV stigma and gender-specific experiences with HIV. Similar topics were discussed in gender-specific groups across both community forums. HIV stigma emerged as a central concern. Participants highlighted the cultural factors that contribute to HIV stigma, emphasizing the consequences of being isolated if someone discloses their HIV status and the secrecy and the taboo around discussing sex. One participant mentioned that “sex can be viewed as sacred and sinful simultaneously.” Specifically, in the women’s small discussion group, issues with gender dynamics were noted. These dynamics often position men as superior to women, with women being unjustly blamed for HIV transmission and related issues. Both men and women accept and often normalize this notion. Women face difficulties in understanding their bodies, expressing themselves, and challenging traditional beliefs. Participants noted that these challenges are further exacerbated when immigrating to more open societies, such as the United States.

Existing prevention strategies were perceived to be ineffective for African immigrants. Participants emphasized the need for community outreach and comprehensive sex education. More strategies should invest in partnerships with the community to ensure more effective outreach efforts. Consistent and widespread culturally sensitive education about HIV/AIDS and other infections within the community was thought to be essential. The role of African men and women in discussing sex with children was explored, revealing a tendency for communication about sex to be led by mothers or other women, with men often absent from these discussions. Women participants expressed a desire for assistance in having open and sex-positive communication with male and female children while also integrating their cultural and religious values. One participant exclaimed, “We do not know how to do it [because] no one did it for us [when we were growing up].” Other strategies discussed included having representatives within the African immigrant community who can openly share their experiences with HIV or related diseases and establishing more social organizations specific to the African immigrant community to address HIV-related issues. Facilitators observed that there was also a lack of basic knowledge on HIV transmission, prevention, and treatment.

Provider Questionnaire: Fifty-three providers completed the questionnaire. In delivering HIV prevention and care services to African immigrants, most providers identified stigma and a lack of cultural appropriateness in services and HIV prevention messaging as significant barriers. Effective strategies used by providers included: ensuring confidentiality, offering open access to publicly funded services and medications, training and employing African immigrants in various roles like Community Health Workers (CHWs) and case managers, conducting outreach in local African immigrant-owned businesses, facilitating community-based testing initiatives, integrating services for a seamless approach (e.g., test and treat strategy), and prioritizing individualized, one-on-one client consultations while addressing broader concerns before focusing on HIV-related aspects. Ineffective interventions for HIV in African immigrant communities involved using existing evidence-based approaches designed for other groups, like African Americans or Caribbean immigrants. Also, aggressive HIV-focused messaging and peer support groups have not proven successful. Similarly, assuming one approach fits all and using English-only campaigns have shown limited effectiveness.

The Tulumbe! Core Circle held two in-person partnership meetings to review data collected from the provider questionnaire and the two community forums, with the aim of developing Comparative Effectiveness Research (CER) topics. Partners also reflected on their experiences as direct care service providers and researchers. It was an iterative process. First, key themes were identified and posted on a board. Then members considered the themes that occurred more frequently and those that were raised by both the community and the providers. Common themes were developed into research topics. Some issues were specific to sub-groups in the African immigrant community—women, men, youth, and families. However, others were cross-cutting (e.g., stigma and communication). See Table 3.

**Table 3:** Five HIV-related research topics developed through a community-led process for addressing HIV among African immigrants.

HIV-related research topic	Description	Population of focus
Improving family communication (parent–child, parent–parent, child–child) about sex and HIV	Discussion about HIV and HIV prevention is not common among African immigrants because there is no dialog between parents and children about sex, safe sex practices and the need for HIV/STD screening. Talking about sex is seen as taboo. In addition, intimate partners rarely discuss sexual feelings and preventive practices such as condom use.	African immigrant families
Empowerment-based interventions to enhance decision-making about risk reduction	African culture regards men as head of the house. Women are expected to be inferior and submissive to men. The power imbalance limits women’s ability to make safer sex decisions. Women need to be empowered to know their bodies, options for sexual health and challenge gender dynamics to protect themselves from HIV and other sexually transmitted diseases.	African immigrant women
Deconstructing masculinity to increase testing, risk reduction and partner communication	The gender power imbalance is a hindering issue that prevents HIV and sex-related discussions among couples. Men are seen as heads of the households giving them the power to make family decisions without regarding their female partner’s inputs. Thus, communication with their partners on HIV is limited. There is a need for men to understand HIV risk reduction and value dialog between their partners to prevent HIV.	African immigrant men
Increase safer sex and testing through education	There is a lack of cultural, youth friendly HIV prevention services tailored to African immigrant youth. Youth are not getting information on HIV prevention and are not engaging in safer sex practices.	African immigrant youth
Reducing HIV stigma	High levels of stigma within the African immigrant community affect HIV/STD transmission, testing, disclosure, access to care and the overall health of African immigrants. HIV stigma is manifested through fear in the community, avoidance of people living with HIV and the belief that HIV infection occurs due to moral failure and is a divine punishment or “curse.” Because of this stigma, African immigrants living with HIV often live in isolation, not telling anyone in their family or community.	African immigrant community

### 4.3 Tier II results

Engagement of Wider Community and Interested Parties: Tulumbe! presented at the Massachusetts Integrated Prevention and Planning Council (MIPPC), a group of consumers, HIV providers, and staff from the Office of HIV/AIDS that provide guidance on HIV prevention and care programs and policy initiatives. The discussion with MIPPC members yielded another topic: health literacy. This refers to the extent to which a person has “the ability to find, understand, and use information and services to inform health-related decisions and actions for themselves and others (27).” A recent study found that African immigrants, including refugees, have low or limited health literacy despite high educational attainment and English proficiency (28). It is well documented that there is a strong relationship between poor health literacy and negative health outcomes which has prompted attention to individual and behavioral interventions to improve individual health literacy (27). The health literacy demands of health care systems and community agencies compound the challenges African immigrants with limited health literacy encounter.

Community Report Back Forum: Thirty-three African immigrants attended the forum. Participants engaged in an interactive process to prioritize research topics and brainstorm research questions. Facilitators guided a discussion with participants to understand the research topic. Using dot voting, all participants prioritized three (3) research topics—(1) reducing HIV stigma in the community, (2) increasing safer sex and testing among African immigrant youth, and (3) improving family communication around sexual health. In small groups, participants were able to further explore the prioritized topics using information sheets, which included (a) narratives and quotes that reflect community perspectives about the research topic (b) state epidemiological data, and (c) findings from the project. Each group received one topic. This structured process facilitated meaningful and informed discussions to identify data gaps and areas requiring research.

Community Questionnaire: In preparation for developing the questionnaire, C.B. facilitated training on CER. During this training partners used the data from the community report-back forum to develop questions that apply to CER and other study designs, such as qualitative exploratory studies. The community questionnaire aimed to gather feedback on prioritized research topics and questions selected during the community report-back forum with the added ‘health literacy’ research topic. Respondents were tasked with ranking the most vital question within each research topic on a scale of 1 (least important) to 5 (most important). Approximately 40 community members completed the questionnaire during a local African event. All were adults representing 16 African countries, primarily Nigeria, Ghana, South Africa, and Kenya. Table 4 outlines the prioritized research topics and questions that were identified through the community engagement process led by Tulumbe! Some of the most popular research questions included, ‘What does disclosure look like for African immigrants living with HIV,’ ‘How are African immigrant youth in the US learning about sexual health,’ and ‘How is sexual health defined, understood, and experienced within African cultural beliefs and understandings?’

**Table 4:** Prioritized research topics and questions.

Research topics	Research questions	Rank
Increase Safer Sex and Testing among African Immigrant Youth	How are African immigrant youth in the US learning about sexual health?	4.4
	How are African Immigrant Youth navigating dual cultural identities (African and African American) and how does that impact decision-making about sexual health?	4.3
	What are the perspectives on same-sex behavior among African Youth?	3.5
Reduce HIV Stigma in the Community	What is the effect of an HIV stigma intervention at the African Health Cup (a soccer tournament held every summer with 12 local African teams) to reduce HIV stigma, improve HIV testing rates, and change HIV-related attitudes?	3.4
	What are the cross-cutting cultural issues related to HIV stigma among African immigrants?	3.8
	What does disclosure (i.e., revealing HIV status) look like for African immigrants living with HIV?	4.3
	What community assets exist to address HIV stigma?	3.9
	How are mental health and substance abuse understood and experienced as factors related to HIV?	3.9
Improve Family Communication about Sexual Health	How is sexual health defined, understood and experienced within African cultural beliefs and understandings?	4.0
	What information around sexual health, sex, and HIV do parents know and what is their motivation around sharing this information with their children?	4.0
	What are the components of a storytelling intervention to improve family communication among African immigrants?	3.6
	What is the shared definition of sex education between parents and youth?	3.3
Increase Health Literacy	How can health providers check for understanding with patients to allow for effective communication and behavior change regarding sexual health?	4.1
	How is LGBTQ (including non-identifying) perceived among African immigrants and how should providers communicate about sexual and gender identity based on community perceptions?	3.8

### 4.4 Dissemination

Partners disseminated the community-defined research priorities to community and academic networks at the end of Tier I and Tier II. Presentations were made at several national and local health and HIV conferences whereas Tulumbe! community members were stipend to attend. Partners also went to different African events, such as the African Festival Lowell, Moroccan Cultural Day, Kenyan Youth Cookout, and African Health Cup to share the research priorities with the community. For dissemination activities, partners created an infographic with the final six health topics. (See Supplementary Image 1. Infographic of Six HIV-related Research Topics).

## 5 Discussion

Despite the significance of community organizations in addressing the critical HIV needs of minoritized and racialized communities, often these organizations are not adequately supported to lead research engagement efforts. With dedicated funding and capacity building, Tulumbe! successfully engaged over 150 African immigrants and more than 50 healthcare providers and researchers through in-person events and questionnaires. Efforts yielded six HIV-related research priorities that were responsive to community needs and critical to addressing HIV among African immigrants in the United States. Our process of developing a research agenda engaged diverse individuals and organizations and was specifically led by African immigrants. The topics and questions were developed for a research agenda but were also applicable to what needs to be accomplished in other areas, such as advocacy, policy, and service delivery.

The creation of a governance document played a pivotal role in unifying our partnership around shared values, guiding principles, and expectations. We demonstrated that being open, appreciating, and understanding that each person at the table has different experiences to offer enriches the collective work of the partnership. This not only fostered collective buy-in and ownership within the partnership but also provided a clear roadmap for our work during both Tier I and Tier II phases. In addition, all partners were committed, fully engaged, and retained throughout the funding cycle. This was evident by meeting attendance, partners taking on specific tasks (i.e., facilitating meetings, leading completion of deliverables), and partners participating in project activities (i.e., facilitating the community forum, disseminating the provider questionnaire).

Because of our partnership’s comradery, we were able to create safe spaces for the community to share and have difficult, realistic, and sometimes personal, conversations about HIV/AIDS. The flexibility in the use of funding further allowed us to actively involve the community in our partnership and offer support for their engagement in a way that fostered leadership. As a community-led group, Tulumbe! served as the initial engagement of members representing the patient population—African immigrants, including their family members, caregivers, community leaders, and the organizations that represent them—and interested parties. Through intentional engagement at various project stages, community members gained confidence in understanding and actively participating in research, thereby enhancing their research literacy.

### 5.1 Challenges with planning and implementation

Tulumbe! had several challenges when planning and implementing the project.

Time Commitment: The project required a significant time commitment from all members. Active engagement included members attending regular meetings, contributing to planning tasks outside of meetings, and leading project activities. Balancing these commitments with other work, school, and family responsibilities was challenging for some members. In addition, the partnership had to manage supporting members who lived far from the central site location, Boston. To minimize these challenges the Program Coordinator maintained ongoing ‘check-ins’ with members, and when possible, Tulumbe! held meetings online. In addition, community members were compensated for travel to project activities and leadership meetings.

Managing Membership: Several members could no longer continue after Tier I. In addition, the governance document was revised in Tier II to include specific language allowing the removal of inactive members. Thus, Tulumbe! had to add members to ensure representation of the community (e.g., youth) and expertise needed (e.g., mental health). This presented an additional layer of complexity because new members joining the partnership needed time to familiarize themselves with the project’s goals, processes, and the unique dynamics of the collaboration. It was important to balance the need for building comradery and effectively integrating the perspectives of these new members while maintaining momentum in implementing the project.

Funding: The unexpected cancelation of Tier III funding had a significant impact on the project, disrupting planned activities and jeopardizing the partnership’s continuity. It was communicated at the last stages of Tier II, so partners had to quickly discuss opportunities to support sustainability. Many funders cater to more traditional research approaches, which makes it difficult to secure the necessary resources for community-driven initiatives that require the time-consuming work of developing the partnership and meaningfully engaging the community. Securing funding, especially for capacity building and community engagement, required Tulumbe! to be creative and strategically identify aspects of the project that were fundable.

Partnership Momentum: Maintaining momentum and enthusiasm among partnership members while waiting for grant funding decisions was difficult. Tulumbe! was able to use this time to disseminate the outcomes of the project.

### 5.2 Sustainability

The development of six community-defined research priorities led to Tulumbe! submitting grants to enhance the sustainability of the community partnership, further explore HIV-related issues among African immigrants, and develop community-driven behavior change and structural interventions. Two proposals were funded (a) a photovoice project developing a digital campaign to address HIV-related stigma funded by Getting to Zero MA (29) and (b) a comparative effectiveness research study to culturally adapt two widely utilized HIV/STI prevention interventions and determine their comparative efficacy in increasing condom and PrEP use among African immigrant Black women funded by PCORI Addressing Disparities (30). In addition, Tulumbe! was cited as part of the 2017–2021 MA Integrated HIV Prevention and Care Plan, which is the framework for the Commonwealth to prevent new HIV infections, reduce health disparities, and improve health outcomes for persons living with HIV infection over the coming 5 years.

## 6 Conclusion

Essential to the HIV response has been that those closest to the problem are often closest to the solution. Providing community-based organizations with the capacity and resources to lead research engagement endeavors can enhance the inclusivity and effectiveness of HIV research. Prioritizing opportunities for African immigrants to lead at all levels of the project—from leadership and decision-making to administering and participating in the engagement activities—created research directions that accurately represent the community’s unique perspectives and lived experiences. In addition, the research priorities identified provided other key entities such as hospitals, health departments, and policymakers with the most current information on the evolving needs of African immigrant communities and the fundamental factors contributing to HIV inequities.

It is imperative that funding allocated for community-led efforts remains flexible to accommodate the time required for establishing a viable research partnership founded on shared values, capacity building, and commitment. Recent initiatives like the National Institutes of Health’s Community Partnerships to Advance Science for Society (ComPASS) Program have started to recognize and support this need (31). However, such opportunities remain scarce. To achieve substantial progress in HIV prevention, treatment, and care, it is vital to bridge the funding gap between community organizations and research entities. Increasing the availability and accessibility of such funding mechanisms has the potential to catalyze meaningful advancements in the field and ensure that community-driven research can thrive and contribute to the broader clinical and public health agenda.

This paper has outlined the processes utilized during two tiers of PCORI P2P funding to actively engage the African immigrant community in identifying research topics of greatest interest to them. The engagement strategies described are not only replicable but also adaptable, offering a blueprint for other contexts where community leadership in defining a research agenda is vital. By documenting and sharing these experiences, we aim to inspire a paradigm shift toward more community-centric research that centers community voices and leadership in shaping health research priorities.

## Data Availability

The raw data supporting the conclusions of this article will be made available by the authors, without undue reservation.
