# Supplementary figures and images for: The Tulumbe! Partnership: a case study in developing a community-led research agenda to address HIV among African immigrants in the United States
Source: Front Public Health. 2024 Sep 20;12:1406397. doi: 10.3389/fpubh.2024.1406397 (PMC11451046; doi:10.3389/fpubh.2024.1406397)

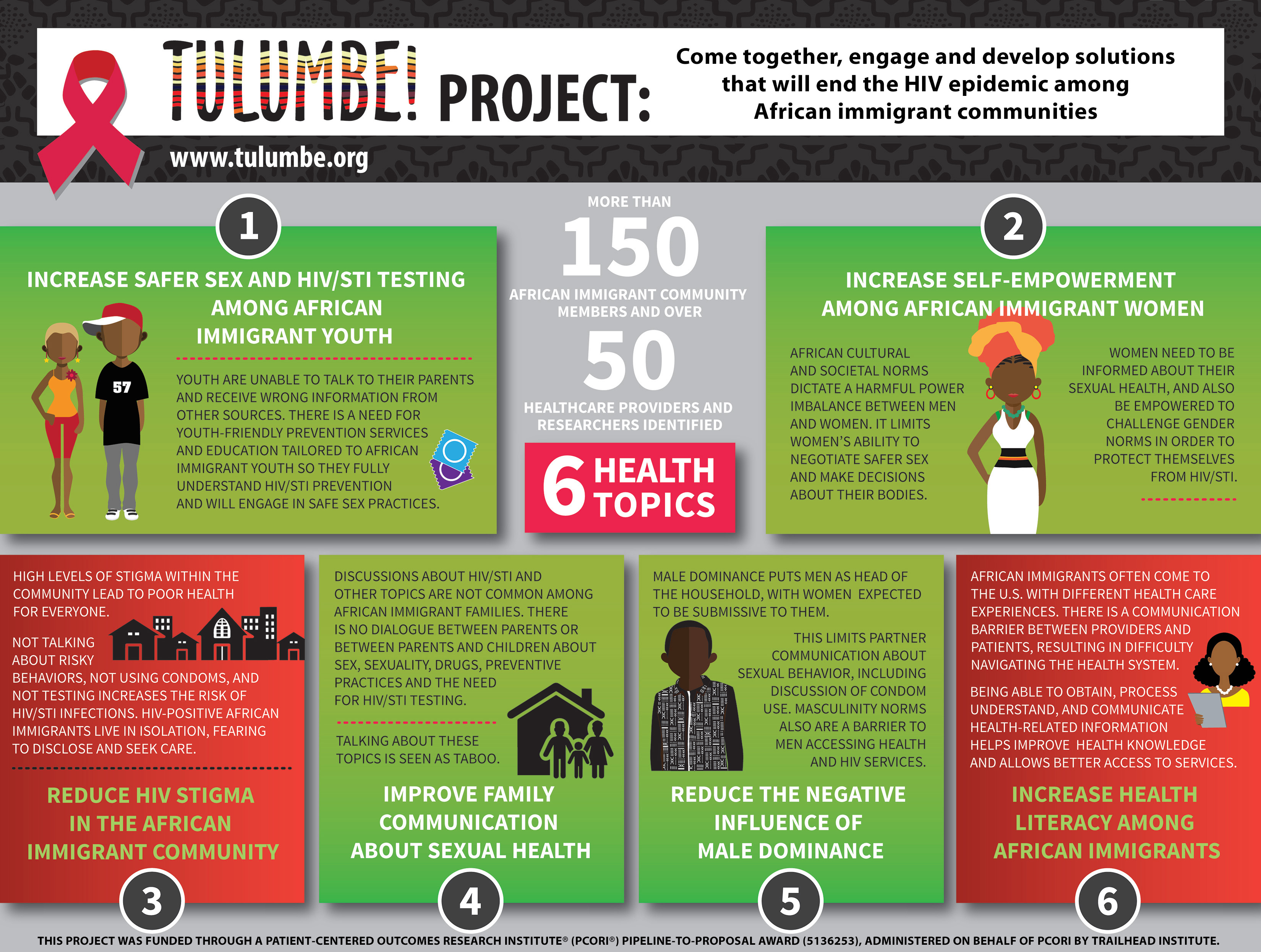

Supplement: Supplementary file 1 [file Image_1.JPEG]
